# Effect of orange almond potato cookies supplementation on the nutritional status of underweight preschool-aged children during COVID-19 pandemic

**DOI:** 10.1371/journal.pone.0266023

**Published:** 2022-04-04

**Authors:** Fatmah Fatmah, Nur Asiah, Etty Rekawati

**Affiliations:** 1 Disaster Management Study Program, School of Environmental Science Universitas Indonesia, Jakarta, DKI Jakarta Province, Indonesia; 2 Faculty of Medicine, Department of Nutrition, YARSI University, Jakarta, DKI Jakarta Province, Indonesia; 3 Faculty of Nursing Universitas Indonesia, Depok, West Java Province, Indonesia; Università degli Studi di Milano, ITALY

## Abstract

Most undernourished preschool-aged children have low hemoglobin and albumin levels, which leads to a higher risk of infections, including COVID-19. This study was designed to determine whether potato almond orange cookies increase weight, hemoglobin, and albumin) in undernourished preschool-aged children during the COVID-19 pandemic. A pre-post intervention study was conducted with 30 subjects during 8 weeks in which hemoglobin and albumin levels were recorded at the beginning and end. Education on balanced nutrition was provided to mothers using leaflets, flipcharts, and videos. The results showed increases in weight (0.4 kg), height (1.98 cm), hemoglobin level (0.1 g/dL), and albumin level (0.1 g/dL) accompanied by a significant increase in weight, height, and the Z-score index for weight for age, whereas those for Hb and albumin levels were not. Energy, carbohydrate, fat, vitamin C, vitamin E, and iron intake increased significantly. Further, there was a significant difference in mothers’ knowledge of balanced nutrition and COVID-19 at the end of the study. Thus, high levels of cookie consumption increased the weight of underweight preschool-aged children. Future studies may wish to consider examining the issue using stunted, wasted, and anemic preschool-aged children as the research subjects.

## Introduction

Non-natural disasters, such as pandemics, have a considerable impact on the lives of people, including preschool-aged children years of age. The COVID-19 pandemic has led to an increase in the morbidity and mortality rates of preschool-aged children not only because of the pandemic, but also due to the increased prevalence of undernutrition [1]. The high number of deaths among preschool-aged children exposed to COVID-19 is suspected to be related to poor nutrition status. The COVID-19 pandemic potentially increases the number of undernourished preschool-aged children because of the economic impact of the pandemic on households. This will further affect under-5 children’s nutritional intakes when there is a limited variety of food provided in the family. There is a possibility that families are now unable to provide food with basic nutrition to preschool-aged children, which then affects the children’s health. The most recent data from UNICEF show that 24 million preschool-aged children are at a higher risk of experiencing undernutrition during the pandemic [2].

Preschool-aged children with malnutrition usually suffer from anemia and low albumin levels that lower immunity, making them more susceptible to COVID-19. Anemia can cause loss of appetite, resulting in reduced weight in children [3,4]. The low level of albumin in preschool-aged children with undernutrition, which is below 3.5 g/dL (hypoalbuminemia), makes them more susceptible to COVID-19 because they have a lower ability to phagocytose and kill bacteria [5,6]. Anemia in preschool-aged children is one of the characteristics of nutritional deficiency, and most preschool-aged children with protein energy malnutrition (PEM) also suffer from anemia. Hypoalbuminemia in undernourished preschool-aged children is associated with an increased risk of infectious diseases, including COVID-19 [7].

During the COVID-19 pandemic, efforts to improve the nutrition status of preschool-aged children with undernutrition and reduce the risk of anemia and hypoalbuminemia may include the provision of supplementary food to their daily food consumption. Cookies made of potato flour, almond flour, orange jam, and eggs are one of the supplementary snacks that can be given to these children. Cookies, with their crunchy and solid texture, are deemed to be a favorite snack for children, and since they do not provide long-term satiety, they are suitable for supplementary food because they will not affect the consumption of the primary food source [8,9].

Orange almond potato cookies made from potato, almond, and orange jam can be given as nutrition supplementation. Potato contains considerable energy and carbohydrates, and thus, it is able to increase weight [10–12]. Almond, with its vitamin E content, can increase the leukocyte count [13]. Finally, orange jam contains hesperidin and serves as an immunomodulator, anti-inflammatory, and antioxidant component [14]. A recent study assessed the efficacy of orange almond potato cookies on 24 underweight older people after 3 weeks of consumption. Increases were seen in weight (0.7 kg), Hb level (0.1 g/dL), and lymphocyte count (1 g/dL) [15]. The objective of the current study was to assess the efficacy of orange almond potato cookies on improving the weight and hematological level (Hb and albumin) of underweight preschool-aged children.

## Materials and methods

### Study design

A pre-post intervention study [16] was applied to 30 preschool-aged children in two selected sub-districts (Pancoran Mas and Sukmajaya) of Depok City at West Java Province who met the inclusion criteria for the study. Ethical clearance was obtained from the Health Research Ethics Commission for Research and Development Agency of the Ministry of Health under Ethical Clearance Number LB.02.01/2.KE.374/2021. All mothers of the study subjects signed informed consent forms before the study began in early June 2021.

### Population and subjects

The population of this study was all children aged 11–57 months who lived in the working area of a community health center at Pancoran Mas Sub-District, Depok City, Indonesia. Sampling was performed using the purposive sampling approach, with the minimum total sample size of the study calculated according to the proportion of the population by testing the difference in proportion hypothesis [17]. Using a previous study’s proportion data, with *p* = 0.0015 [18], 95% power, and two-sided 5% significance test, the sample size was determined to be 35.

This study began with the nutrition status screening of 65 preschool-aged children with undernutrition in Depok, Mampang, and Rangkapan Jaya Lama urban villages, Pancoran Mas Sub-district, Depok City, on June 29, 2021. Based on the results of the screening, 46 preschool-aged children who met the inclusion criteria were identified. They were aged 12–57 months and included both boys and girls living in one of the three urban villages; the participants had poor nutritional status with a Z-score of W/A less than minus 2 standard deviations (SD) [19], and they were not suffering from any infectious or chronic diseases. However, at the time of informed consent signing with the mothers, which was followed by blood sampling for hemoglobin (Hb) and albumin tests, 11 mothers refused to have their children’s blood taken because they were worried that their children would experience fever, the father did not allow blood sampling, or the children were ill.

### Cookie supplementation

From June to August 2021, for 8 weeks, 30 preschool-aged children who participated in the full study were given 50 g of orange almond potato cookies every day. Every 50 g of orange almond potato cookies contained 237.7 kcal of energy, 28.8 g of carbohydrates, 3.5 g of protein, 12.1 g of fat; 0.25 mg of vitamin C, 2.5 mg of vitamin E, 1.5 mg of Fe, and 1.1 mg of Zn (Table 1). Cookies were made using a mixer, digital oven, baking sheet, cookie dough cutter, and digital kitchen scale. The ingredients of the cookies comprised potato flour, almond flour, orange jam, eggs, refined white sugar, butter, and pandanus/strawberry paste/flavoring. The cookies were made from 1–2 kg of potato starch, 0.5–1 kg of almond flour, and 1–2 kg of orange jam. The ratio of these ingredients was 2:2:1. The cookies were prepared freshly every week, each time with different batches of product to minimize the loss of vitamins and minerals during the storage process. The orange almond potato cookies were analyzed for macro- and micronutrients at Saraswanti Genetech Laboratory of Bogor City using proximate test analysis [20].

**Table 1:** Nutrient content per 50 g of almond potato orange cookie.

Type of cookie	Energy (kcal)	Carbohydrate (g)	Protein (g)	Fat (g)	Vitamin C (mg)	Vitamin E (mg)	Fe (g)	Zn (g)
Almond potato orange	237.7	28.8	3.5	12.1	0.25	2.5	1.5	1.1

Source: Saraswanti Laboratory Bogor, 2021.

### Nutritional status measurement

In terms of nutritional status of the preschool-aged children, to meet the inclusion criteria, study subjects needed to have Weight for Age (W/A) Z-score of less than minus 2 SD; this was determined via the measurements of weight and height. The measurement results were entered into the World Health Organization (WHO) Anthropometry to obtain the Z-score for W/A. Secondary data of the subjects were obtained from the sub-district and urban village community health centers. Mid-upper arm circumference (MUAC) was measured before the start of cookie consumption in all subjects. Blood sampling was performed before and after the study (on day 60) to measure the Hb and albumin levels. A digital scale with a precision of 0.1 kg was used for weighing. The subjects’ height was measured with a microtoise with a precision of 0.1 cm. The midline band was used to measure the MUAC of the subjects at the beginning of the study.

### Baseline and end-line data collection and daily food consumption records

Independent variables measured in baseline and end-line data were socio-demographic characteristics (marital status of mothers of preschool-aged children, maternal age, maternal occupation, paternal occupation, people living in the same house, gender of child under 5, age of child under 5, number of preschool-aged children in the family, and the person who most often takes care of the preschool-aged children), knowledge of balanced nutrition for preschool-aged children, knowledge of COVID-19, access to daily grocery shopping, and feeding practices (daily variation of nutritious food during the COVID-19 pandemic and Food Frequency Questionnaire on food as a source of carbohydrates, protein, fat, vitamins, and minerals). The purpose of collecting baseline and end-line data was to determine the level of change in mothers of under-5 children’s knowledge of balanced nutrition and COVID-19, as well as to identify the under-5 children’s feeding behaviors. The 24-hour food recall form was used by trained enumerators to record data on subjects’ daily food consumption for 8 weeks. The recording of daily food consumption for preschool-aged children was carried out via home visits at the beginning, middle, and end of the week for 8 weeks (a total of 24 days of daily food records for each subject) in conjunction with the recording of the cookie distribution. The cookie distribution form collected data on the number of cookies given to mothers of preschool-aged children each time the enumerator visited, the number of cookies consumed, and the number of cookies remaining. Education on balanced nutrition for preschool-aged children and COVID-19 was provided three times during the study using flipcharts, leaflets, and videos developed by the research team.

### Monitoring of adherence to cookie consumption and health status

Adherence to cookie consumption by child under 5 was monitored using a distribution record form during home visits three times a week and measurements of anthropometric data at the integrated service post (*posyandu*) two times a week during the study. At each home visit, data on food consumption the day before the visit, cookies distribution, number of remaining cookies, complaints or side effects after cookie consumption, and current health status were recorded. At the first home visit, it was revealed that some preschool-aged children did not like their cookies, so they were eaten by other family members. However, after the mother was motivated by the enumerators to keep persuading their preschool-aged children to eat the cookies to increase their child’s weight, all preschool-aged children began to like eating the cookies. During the 8-week study, four preschool-aged children suffered from mild illnesses, such as fever, cough, and cold. Although they did not test positive for COVID-19, both their appetite and their consumption of cookies decreased during their episode of illness.

### Data analysis

Univariate analysis was performed to obtain the mean values of sociodemographic characteristics, knowledge of balanced nutrition for preschool-aged children with Protein Energy Malnutrition, knowledge of COVID-19, feeding practices, anthropometric characteristics of preschool-aged children (weight, height), and immunity status (Hb and albumin levels) using SPSS Version 13 software. The results of this analysis were then tabulated. The pre- and post-study anthropometric data of the children were processed using the WHO Anthro Program 3.2 [21]. The 8-week food consumption data were analyzed using the Nutri Survey Program [22]. The purpose of the consumption data analysis was to assess the intake of macronutrients (energy, carbohydrates, proteins, fats) and micronutrients (vitamin C, vitamin E, Fe, and Zn) before and after the study. Bivariate analysis with a statistical paired *t*-test was used to assess changes in mean weight, height, Hb status, albumin level, macronutrient intake, and micronutrients before and after the study. Meanwhile, repeated measurements of weight (4 times in 8 weeks) were analyzed using ANOVA.

## Results

**Fig 1:**
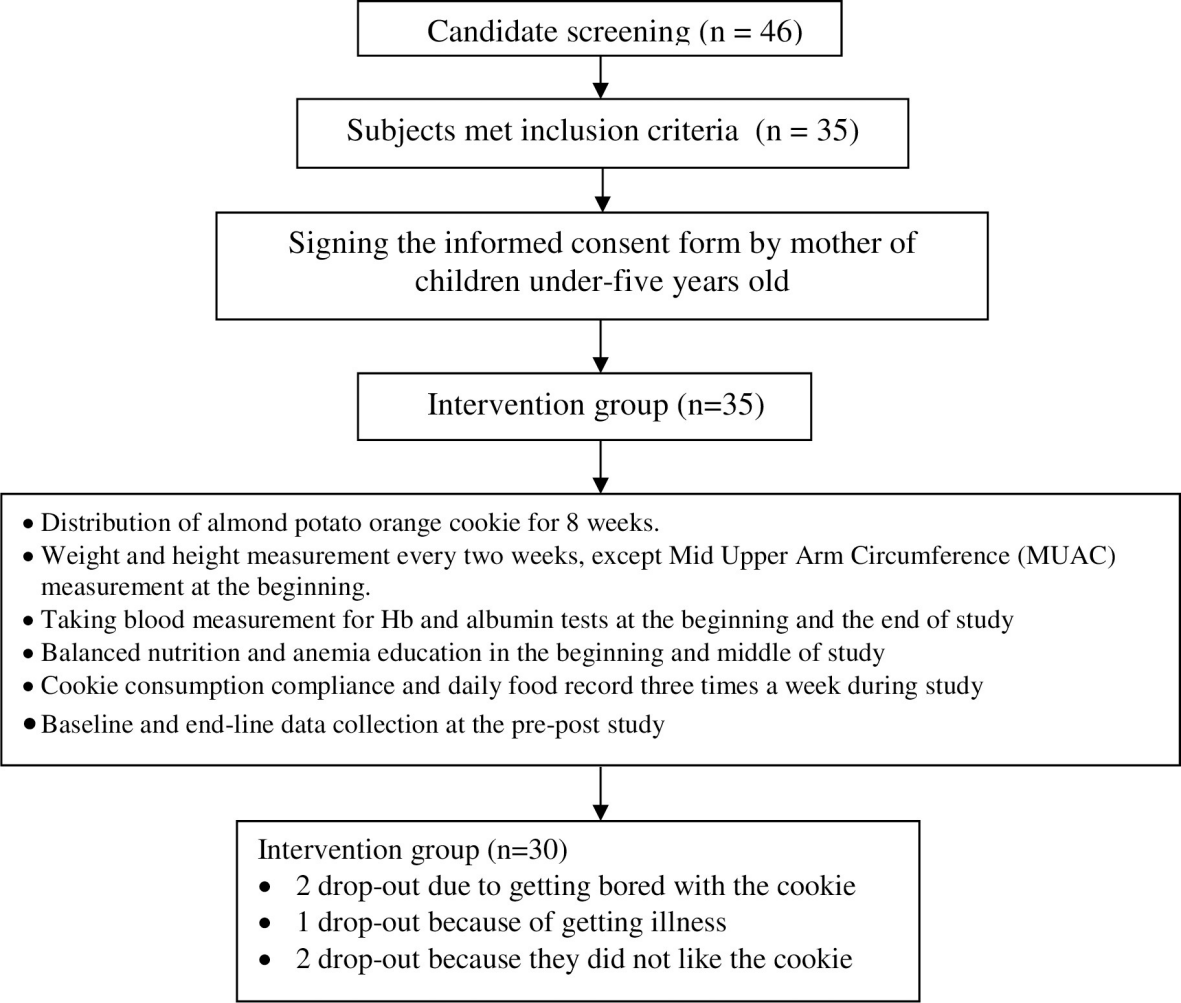
Research scheme.

There were 30 preschool-aged children who met the inclusion criteria and participated in the study. Five subjects dropped out of the study because they were seriously ill and needed to be hospitalized, became bored with the cookies, or did not like the sweet taste of the cookies because of a preference for savory snacks (Fig 1). When the preschool-aged children were asked about their impressions of the cookies during the home visits, all of them stated that they liked them because they were delicious. The cookies were suitable for preschool-aged children because of their sweet taste and soft texture. In addition, there were no side effects found, such as having difficulty defecating or nausea. However, some mothers of preschool-aged children said that after their children ate the cookies, they felt full and refused to have lunch or dinner. In contrast, some children felt that their appetites increased.

All mothers of preschool-aged children who participated in the study were married. Most of these mothers were in the age range of 30–39 years, with some being over 40 years old. Fifty percent of these mothers had completed their primary and secondary education and almost all of them did not work outside the home. The fathers were mostly private employees, with laborers and traders having the same proportion as those who were unemployed. More than three-quarters of all preschool-aged children still lived with their families (Table 2). Girls comprised the majority of the subjects, with most participants being in the age ranges of 24–35 months and 48–57 months. When asked about birth certificates to ascertain the children’s age, nearly three-quarters of the children had birth certificates, and these could be shown to the enumerator by the mothers upon request. Almost all mothers of these preschool-aged children stated that they had only one child when asked about the number of preschool-aged children they had at the time of the study. Mothers were the ones who most often took care of their preschool-aged children at home, and almost all preschool-aged children in this study lived with their mothers, with a small percentage being taken care of or living with their father, grandmother, or aunt (Table 2).

**Table 2:** Socio-demography and anthropometry characteristic of subjects.

Variable	n%	Mean *±* SD (Min–Max)
*Characteristic of family*		
Marital status		
Married	30	100.0
Widow/widower	0	0.0
Mothers’ age (y.o)		33.3 ± 6.2 (20–47)
20–29	6	20.0
30–39	19	63.3
40–46	5	16.7
Mothers’ education		
Graduated from junior high school or less	17	56.7
Graduated from senior high school	10	33.3
Academy / bachelor	3	10.0
Working status of mother		
No	28	93.3
Yes (trader)	2	6.7
Working type of father		
Private	9	30.0
Trader	6	20.0
Laborer	6	20.0
Government employee	3	10.0
No job	6	20.0
Residence status		
Family (father, mother, children)	22	73.3
Join with another family	8	26.7
*Characteristic of children under-five*		
Gender		
Female	17	56.7
Male	13	43.3
Age (months)		
11–23	7	23.3
24–35	8	26.7
36–47	7	23.3
48–57	8	26.7
Ownership of birth certificate		
Yes	22	73.3
No	8	26.7
Number of children under-five in family		
One	27	90.0
Two	3	10.0
The person who most often takes care of toddlers		
Mother	21	70.0
Father and mother	4	13.3
Mother and grandmother/aunt	5	16.7

Table 3 illustrates the differences in the pre- and post-study anthropometric characteristics of the subjects. There were differences in weight, height, and Z-score of W/A before and after the study, showing increases in those variables (*p* < 0.01). However, Hb and albumin were not significantly correlated with the consumption of cookies in all subjects at post study. At the end of the study, there was a significant difference in the macronutrient intake, including energy, carbohydrates, fat, vitamin E, and iron, when compared with the baseline. There was also a significant difference (*p* < 0.01) in the balanced nutritional knowledge and knowledge of COVID-19 after subjects were given supplementation of orange almond potato cookies for 8 weeks between the pre- and post-study.

**Table 3:** Mean changes in anthropometric characteristics of children under-five, knowledge of nutrition and COVID-19, feeding practices of children under-five, and macro and micro nutrient intake at pre-post study.

Variable	*Mean ± SD (Min-Max)		p-value
	Pre/before	Post/after	
*Anthropometry characteristic*			
Weight (kg)	10.6 ± 1.9 (6.5–14.0)	10.9 ± 1.9 (7.5–14.4)	**0.01
Height (cm)	85.9 ± 9.9 (62.2–101.6)	87.9 ± 10.0 (64.5–104.0)	**0.01
Z-score (W/A)	-2.2 ± 0.8 (-3.8–0.4)	-1.65 ± 1.2 (- 4.5–2.3)	**0.01
Hemoglobin (g/dL)	12.3 ± 1.6 (7.4–14.8)	12.2 ± 1.7 (8.1–14.5)	0.42
Albumin (g/dL)	4.8 ± 0.5 (3.6–6.0)	4.9 ± 0.5 (4.0–5.9)	0.35
Score of balanced nutrition knowledge	57.0 ± 15,5 (36.4–90.9)	89.4 ±13.7 (54.6–100)	**0.01
Score of COVID-19 knowledge	73.7 ± 13.5 (33.3–88.9)	81.9 ± 11.8(44.4–88.9)	**0.01
*Macronutrient intake*			
Energy (cal)	944.9±212.2 (493.0–1393.6)	1117.3± 211.4 (620.0–1434.3)	**0.01
Carbohydrate (g)	115.5 ± 34.6 (52.0–198.7)	145.3 ± 33.8 (84.1–211.9)	**0.01
Protein (g)	34.4 ± 10.3 (15.0–57.2)	34.6 ± 7.3 (16.8–44.8)	0.88
Fat (g)	37.8 ± 10.5 (16.3–66.0)	43.8 ± 9.4 (25.0–60.0)	**0.01
Micronutrient intake			
Vitamin C (mg)	21.1 ± 23.5 (2.0–87.8)	22.0 ± 15.2 (3.9–63.2)	0.87
Vitamin E (mg)	3.3 ± 3.8 (0.0–22.4)	4.8 ± 3.5 (1.6–22.4)	**0.01
Iron/ Fe (g)	4.5 ± 2.6 (1.4–11.9)	6.0 ± 2.2 (2.0–11.9)	**0.01

* Dependent t test.

**p < 0.05.

Changes in weight, height, and Z-score of W/A in relation to socio-demographic characteristics, under-5 children’s feeding practices, the cookie consumption rate, the adequacy level of % Recommended Dietary Allowance (RDA) for macro-micronutrients, knowledge of balanced nutrition for preschool-aged children, and knowledge about COVID-19 at the end of the study are presented in Table 4. Under-5 children’s feeding practice and the consumption level of orange almond potato cookies affected weight gain in preschool-aged children. The age of the mothers of preschool-aged children, the under-5 children’s feeding practices, and the level of consumption of orange almond potato cookies affected the increase in the Z-score of W/A at the end of the study.

**Table 4:** Changes in weight, height, and Z-scores W/A based on socio-demographic characteristic, feeding practices, cookies consumption rate, adequacy level of % RDA for macro-micro nutrients, knowledge of balanced nutrition, and knowledge on COVID-19 at the end of study.

Variable		n	*Weight difference (kg)			p	*Height difference (cm)			p	*Z Score W/A difference			p	*Hemoglobin difference (g/dL)			p	*Albumin difference (g/dL)			p-value
			Mean	±	SD		Mean	±	SD		Mean	±	SD		Mean	±	SD		Mean	±	SD	
Feeding practice of children under-five at pre-study																						
	Not good (< = mean)	19	0.45	±	0.55	0.564	1.97	±	0.67	0.630	0.30	±	0.43	0.353	-0.04	±	0.87	0.640	-0.04	±	0.33	0.822
	Good (> mean)	11	0.35	±	0.34		1.81	±	0.99		0.50	±	0.61		-0.18	±	0.73		-0.06	±	0.30	
Feeding practice of children under-five at post-study																				±		
	Not good (< = mean)	13	0.18	±	0.45	**0.01	1.97	±	0.82	0.740	0.06	±	0.34	**0.01	-0.08	±	0.87	0.771	-0.05	±	0.31	0.892
	Good (> mean)	17	0.59	±	0.44		1.87	±	0.78		0.60	±	0.49		-0.17	±	0.63		-0.06	±	0.33	
Level of cookie consumption during study (g)																				±		
	Poor (< = 2,550)	15	0.21	±	0.43	**0.01	2.06	±	0.89	0.315	0.15	±	0.42	**0.01	-0.12	±	0.81	0,982	-0.12	±	0.30	0.243
	Good (> 2,500)	15	0.61	±	0.45		1.77	±	0.67		0.59	±	0.49		-0.11	±	0.78		0.01	±	0.31	
Adequacy level of macronutrient RDA %																				±		
	*Energy*																			±		
	Poor (< 70%)	6	0.50	±	0.40	0.629	1.37	±	0.39	0.055	0.47	±	0.24	0.609	0.38	±	0.65	0.080	0.15	±	0.16	0.070
	Good (> = 70%)	24	0.39	±	0.50		2.05	±	0.80		0.35	±	0.55		-0.24	±	0.77		-0.10	±	0.32	
	*Carbohydrate*																			±		
	Poor (< 70%)	19	0.43	±	0.54	0.789	1.87	±	0.72	0.723	0.32	±	0.47	0.528	0.21	±	0.60	0.090	0.08	±	0.14	0.256
	Good (> = 70%)	11	0.38	±	0.37		1.98	±	0.93		0.45	±	0.58		-0.67	±	0.77		-0.28	±	0.39	
	*Protein*																					
	Good (> = 70%)	30		-				-				-				-				-		
	*Fat*																					
	Poor (< 70%)	3	0.50	±	0.61	0.747	1.50	±	0.50	0.346	0.37	±	0.25	1.000	0.47	±	0.99	0.178	0.07	±	0.15	0.487
	Good (> = 70%)	27	0.40	±	0.48		1.96	±	0.80		0.37	±	0.53		-0.18	±	0.75		-0.07	±	0.32	
Adequacy level of micronutrient RDA %																						
	*Vitamin C*																					
	Poor (< 70%)	22	0.35	±	0.45	0.203	1.91	±	0.77	0.962	0.31	±	0.53	0.274	-0.11		0.78	0.973	-0,04		0.30	0.721
	Good (> = 70%)	8	0.60	±	0.55		1.93	±	0.89		0.54	±	0.42		-0.13		0.85		-0,09		0.35	
	*Vitamin E*																					
	Poor (< 70%)	14	0.30	±	0.49	0,232	2.00	±	0.82	0.581	0.28	±	0.56	0.394	-0.14		0.76	0.903	-0,03		0.32	0.688
	Good (> = 70%)	16	0.51	±	0.47		1.84	±	0.78		0.45	±	0.46		-0.10		0.83		-0.08		0.31	
	*Iron/Fe* (g)																					
	Poor (< 70%)	12	0.30	±	0.49	0.298	2,21	±	0.75	0.094	0.19	±	0.46	0.114	-0.19		0.88	0.676	-0.11		0.36	0.434
	Good (> = 70%)	18	0.49	±	0.47		1,72	±	0,77		0.49	±	0.51		-0.07		0.73		-0.02		0.27	
Level of improving balance nutrition knowledge																						
	Poor	13	0.38	±	0.49	0.723	1.84	±	0.80	0.656	0.42	±	0.58	0.675	-0.04	±	0.91		-0.03	±	0.34	
	Good	17	0.44	±	0.49		1.97	±	0.79		0.34	±	0.45		-0.18	±	0.69	0.640	-0.07	±	0.29	0.732
Level of improving COVID-19 knowledge																						
	Poor	12	0.43	±	0.59	0.856	1.79	±	0.88	0.498	0.49	±	0.56	0.283	-0.15	±	0.83		-0.03	±	0.31	0.777
	Good	18	0.40	±	0.40		1.99	±	0.73		0.29	±	0.46		-0,.9	±	0.78	0,857	-0.07	±	0.32	

*Independent t-test.

** p<0.05.

## Discussion

The COVID-19 pandemic has had a major impact on preschool-aged children, and one of its effects has been the increased prevalence of preschool-aged children with undernutrition because of the economic impact experienced by their families. A reduction in family income has reduced the ability of the family to buy nutritious food, reducing the nutritional intake of preschool-aged children. A non-natural disaster, such as the COVID-19 pandemic, can potentially result in food and nutrition emergencies and its impact to address the nutritional challenges in preschool-aged children. There is a critical period to address nutritional challenges in preschool-aged children when natural disasters and non-natural disasters occur. However, the handling of the malnutrition problem in both conditions among preschool-aged children is almost the same. To anticipate the problem in the context of COVID-19, in this study, orange almond potato cookies were created as food that would be supplementary to the daily food consumption of preschool-aged children.

The *posyandu* team consisted of cadres and nutritionists of primary health care, and the research team conducted the research for 8 weeks. However, the research team reported that supervising the children weekly required considerable time because of the mothers’ busy schedules. Sometimes, the children’s mothers were not at home when the research team visited their house. According to mothers, the variability of the color, flavor, and shape of the cookies fed to children was observed to improve the children’s appetites. Low variation of cookies in the children’s diets can cause low food intake among children. However, in the supervision activities, the team found that mothers experienced several obstacles, such as their children becoming bored quickly and children’s loss of appetite because of illness, resulting in a lack of desire to eat cookies. Mothers sometimes added sugar, ice cream, milk, and pudding to make the cookies taste better.

The present study indicated that the orange almond potato cookies as supplementary feeding for 8 weeks effectively improved the weight (0.4 kg), height (1.98 cm), Hb level (0.1 g/dL), and albumin level (0.1 g/dL) of underweight children. The improvement of weight in the current study was lower than Lubis et al. (2020) and Herawati et al. (2018) [23,24]. These variances likely emerged because of differences in location, ingredients in the supplementary foods, and location setting. In addition, the findings showed that Hb and albumin levels did not differ significantly after the intervention study. This may have been caused by the short time of the nutrition intervention implementation, the limited number of subjects, and the fact that the cookies were not fortified with iron, vitamin C, or Zn. The mean pre-study Hb level, which was categorized as normal, may also have contributed to the lack of significant difference before and after the orange almond potato cookies were added to the diet. Indeed, the cookies program should be undertaken for 90 days for the best results [25]. The main result of the present study was that orange almond potato cookies were demonstrated to trigger increases in weight (0.4 kg), height (1.98 cm), Hb level (0.1 g/dL), and albumin level (0.1 g/dL). The W/A indicator is one measurement of nutritional status indicating underweight; it reflects current and acute malnutrition [26,27].

Some studies [23,28–30] reported that supplementary feeding programs improved the nutritional status of underweight children. The current study showed no significant difference in hematological status (Hb and albumin). However, Nazni et al. (2010) and Owino et al. (2007) reported that supplementation of energy-dense foods may improve Hb concentration [31,32]. In contrast, Widodo et al. (2015) reported that biscuit formula based on cork fish and brown rice for 90 days could improve the serum albumin of underweight children [29]. Low levels of albumin are usually found in cases of undernourished preschool-aged children and are manifested in the form of marasmus, kwashiorkor, and marasmus kwashiorkor and undernutrition. Reduced intake of high-protein foods can decrease protein synthesis, leading to hypoalbuminemia. Hence, the hypoalbuminemia of undernourished preschool-aged children can be assessed in terms of their inadequate dietary protein intake [33]. All subjects who consumed orange almond potato cookies for 2 months in this study had adequate levels of protein and zinc intake (≥70% RDA). The pre-post mean albumin was also relatively normal. The two indicators of adequacy of protein consumption and appropriate blood albumin levels are thought not to have affected the albumin levels before and after cookie consumption.

The consumption level of orange almond potato cookies and good feeding practices for preschool-aged children influenced changes in the weight and Z-score of W/A in this study based on the comparison of the pre-post study values. Good feeding practice includes no change in the pattern of breastfeeding or formula intake during the COVID-19 pandemic; the ability of mothers to always provide a variety of food that include carbohydrates, animal protein, vegetable protein, and vegetables at every meal, although not every day; and the frequency of preschool-aged children eating the types of nutrient sources of carbohydrates, proteins, fats, vitamins, and minerals being categorized as every day (7 days/week) and/or sometimes (3–5 times/week). Most mothers of preschool-aged children in this study applied good feeding practice, which was evidenced from the improvement from undernutrition status to normal nutrition status. In contrast, this was not the case with the study of the nutritional status of preschool-aged children in Sukoharjo District, Central Java Province Indonesia, which showed no relationship between feeding practice and the nutritional status of these children [34].

Energy, carbohydrate, fat, vitamin C, vitamin E, and Fe intakes showed significant differences in the treatment group, but no effect on weight, height, Z-score of W/A, Hb, or albumin was observed. This was because the intake level of these five nutrients was still low, reaching below the adequate level of 70% RDA, except for protein and zinc, which was already good. Orange almond potato cookies were not fortified with iron, vitamin C, zinc, or high-protein foods, such as cork fish, to increase mean Hb and albumin at the end of the study. By consuming 50 g of orange almond potato cookies every day, a child under 5 can meet 17–17.5% RDA of energy, 13–13.4% RDA of carbohydrates, 14–17.5% RDA of protein, 24.2–26.9% RDA of fat, 0.6% vitamin C, 35.7–41.7% RDA of vitamin E, 15–21.4% RDA of Fe, and 22–36.7% RDA of zinc [35]. The interaction of vitamin C with iron in increasing blood Hb is that vitamin C can accelerate Fe absorption by converting it to Fe^+3^, which is more easily absorbed by the body [36].

Knowledge of balanced nutrition and COVID-19 was significantly different after the study when compared with the pre-study level. Although the knowledge of balanced nutrition and COVID-19 did not affect the increase in weight, height, Z-score of W/A, Hb level, or albumin level at the end of the study, but a sufficient level of balanced nutritional knowledge tended to lead to a slightly larger difference in weight, height, and Z-score of W/A than a lower level of knowledge did. Some studies reported that supplementary feeding with nutrition education improved the nutritional status of underweight children [23,37–39]. This finding contradicts the finding of a study on the influence of nutrition counseling on under-5 children offered to mothers of undernourished preschool-aged children in Medan, which proved the significant effect of counseling on weight for age in preschool-aged children with *p* < 0.05 [40]. Nutrition education is effective in increasing nutritional knowledge and eventually improving nutritional status. Several previous studies have also shown the effectiveness of nutrition education on the knowledge of mothers of preschool-aged children and the nutritional status of these children.

## Conclusions

The provision of orange almond potato cookies as supplementary food for preschool-aged children with undernutrition affects weight gain and nutritional status toward normal values. Intakes of energy, carbohydrates, fats, vitamin C, vitamin E, and Fe, as well as knowledge of balanced nutrition and COVID-19 in the treatment group, were significantly different before and after supplementation with orange almond potato cookies. The level of orange almond potato cookies and feeding practice resulted in increased weight and Z-scores of W/A among preschool-aged children with undernutrition. Nutritional supplementation via Oral Nutrition Supplementation in the form of orange almond potato cookies needs to be accompanied by balanced nutritional education activities to improve adherence to consumption. With the slight increase in weight and no change in hematological variables in the study, it is suggested that a similar study using larger cookie servings of 100 grams daily, a larger sample size, a longer period of study, and inclusion of a control group as a comparison to the treatment group should be performed to achieve maximum results with an expected output of an even greater increase in weight and significant changes in Hb and albumin levels via supplementation with orange almond potato cookies.
